# Robotic-assisted single-port choledochal cyst radical surgery in children – a case series study

**DOI:** 10.1097/MS9.0000000000003712

**Published:** 2025-09-09

**Authors:** Jianglong Chen, Yuru Zhang, Shan Lin, Shaohua He, Yufeng He, Guangxu You, Jiaxing Xu, Di Xu

**Affiliations:** a Shengli Clinical Medical College of Fujian Medical University, Fuzhou, Fujian Province, China; b Department of Pediatric Surgery, Fuzhou University Affiliated Provincial Hospital, Fuzhou, Fujian Province, China; c College of Stomatology, Changsha Medical University

**Keywords:** case series study, choledochal cyst, da Vinci Xi, pediatric minimally invasive surgery, single port

## Abstract

**Background:**

We pioneered the use of the da Vinci Xi system’s robotic-assisted single-port technology for pediatric choledochal cyst resection. We assessed the feasibility and safety of this new surgical approach.

**Methods:**

Our study included five pediatric patients diagnosed with choledochal cyst. We described the surgical procedures in detail. We established the single port through the umbilicus. The jejunal Roux limb and intestinal anastomosis were performed outside the abdomen through the single port. Then, we removed the gallbladder and cyst and performed biliary-intestinal anastomosis using the robotic system. We collected characteristic data and perioperative data.

**Results:**

All surgeries were successful. The total operative time ranged from 180 to 232 minutes, and the robotic operative time ranged from 60 to 90 minutes. The fasting time ranged from 3 to 7 days, and the hospitalization time ranged from 7 to 14 days. We did not observe any complications, including blood vessel, intestine, or pancreas injury, intra-abdominal or wound infection, cholangitis, or biliary fistula.

**Conclusion:**

The robotic-assisted single-port technology is safe and effective for pediatric choledochal cyst resection using the da Vinci Xi system. However, further studies with larger patient populations are needed to provide more information on long-term complications and the learning curve.

## Introduction

Choledochal cyst is a congenital anomaly of the bile ducts in children. The primary surgical treatment for choledochal cysts involves radical surgery, which includes complete cyst excision and Roux-en-Y hepaticojejunostomy anastomosis. This procedure can be performed using either open or laparoscopic techniques. Minimally invasive surgery offers significant advantages for pediatric patients[1]. Robotic-assisted pediatric choledochal cyst excision was first reported by Woo R in 2006 and has since become widely adopted[2]. Another approach to minimize trauma in minimally invasive surgery is single-incision surgery, which has gained popularity due to its cosmetic benefits[3]. Currently, single-port laparoscopy and da Vinci robot-assisted multi-port laparoscopic surgery are employed in pediatric procedures, including the radical resection of choledochal cysts. However, robot-assisted single-port radical resection of choledochal cysts has not yet been applied in pediatric patients. And the Da Vinci SP, a single-port platform, is not suitable for pediatric patients due to their limited abdominal or pelvic capacity[4]. In this study, we utilized the Da Vinci Xi system to perform single-port surgery for pediatric choledochal cyst excision and Roux-en-Y hepaticojejunostomy anastomosis.

## Methods

This retrospective study included five pediatric patients diagnosed with choledochal cysts, all of whom underwent robotic-assisted single-port choledochal resection. The inclusion criteria were patients with choledochal cysts, excluding those with recurrent cholangitis, potential adhesion inflammation around the common bile duct, and confirmed anatomical variations preoperatively.

The studies involving human participants were reviewed and approved by. Written informed consent was obtained from the legal guardians or next of kin of the participants.

**Figure 1.**
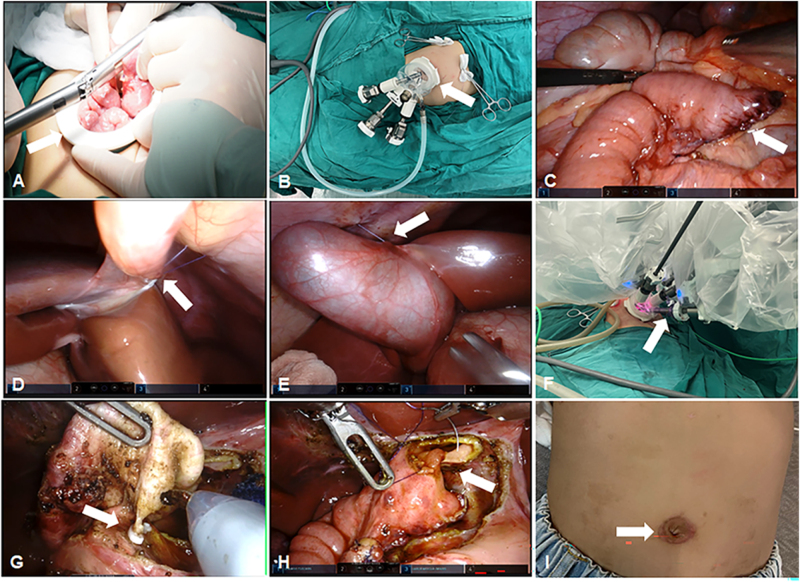
Robotic-assisted single-port choledochal cyst radical surgical procedures. (A) Jejunoenteroenterostomy was performed extracorporeally through the umbilical single port (the white arrow). (B) The single port was shown before the robotic instruments docking (the white arrow). (C) The jejunal hepatic limb is placed at the hilum of the liver through the transverse mesocolon (the white arrow). (D) and (E) The round hepatic ligament and the base of the gallbladder were suspended at the abdominal wall (the white arrow). (F) Then da Vinci Xi system was docked to the patient (the white arrow). (G) The distal end of the common bile duct was properly ligated (the white arrow). (H) The hepatic branch of the jejunal loop is anastomosed with the common hepatic duct of the hilar part of the liver (the white arrow). (I) The scar was shown after 1 month (the white arrow).

Under general anesthesia and endotracheal intubation, a ureteral catheter and a gastric catheter were placed. An arc incision was made around the umbilicus, and a single port was inserted through this incision. The jejunoenteroenterostomy was performed extracorporeally through the umbilical single port (Fig. 1A). The jejunum, approximately 15–20 cm distal to the duodenal Treitz ligament, was brought out through the umbilical incision, and the jejunal Roux limb and intestinal anastomosis were performed outside the abdomen. An artificial pneumoperitoneum was established. The single port was positioned before the robotic instruments were docked (Fig. 1B). The jejunal Roux limb was placed at the hepatic hilum through the transverse mesocolon (Fig. 1C). Patients were positioned in a supine and head-high left lateral tilt. The ligamentum teres hepatis and the gallbladder fossa were suspended to the abdominal wall (Fig. 1D, E). The da Vinci Xi system was then docked to the patient (Fig. 1F).

The gallbladder and choledochal cyst were resected, and the distal end of the common bile duct was appropriately ligated (Fig. 1G). The jejunal Roux limb was anastomosed with the common hepatic duct at the hepatic hilum (Fig. 1H). The gallbladder and choledochal cyst were removed through the single port. The incision was sutured, and the scar is shown 1 month postoperatively (Fig. 1I). The robotic procedure is demonstrated in the supplementary video, http://links.lww.com/MS9/A929.

This case series has been reported in line with the PROCESS Guideline .

## Results

All patients underwent robotic-assisted single-port choledochal resection for choledochal cysts. Each surgery was completed successfully without conversion to open surgery or alternative procedures. The patient cohort comprised one male and four female patients, aged between 2 and 12 years. All patients had type Ia choledochal cysts. The total operative time ranged from 180 to 232 minutes, with the robotic operative time ranging from 60 to 90 minutes. Fasting periods varied from 3 to 7 days, and hospitalization durations ranged from 7 to 14 days (Table 1). One patient (No. 3) had recurrent cholangitis preoperatively and underwent a biliary ostomy 3 months prior; this patient’s fasting and hospitalization times were 7 and 14 days, respectively. No complications, such as vascular, intestinal, or pancreatic injuries, intra-abdominal or wound infections, cholangitis, or biliary fistulas, were observed in the study. The blood loss volumes were estimated using a small moistened gauze, as the losses were minimal.

**HIGHLIGHTS**

- We successfully applied robotic-assisted single-port technology using the da Vinci Xi system for pediatric choledochal cyst resection for the first time.
- The robotic-assisted single-port technology was found to be safe and effective for pediatric choledochal cyst resection when using the da Vinci Xi system.
- This technology offers a reliable approach and valuable insights for the future application of single-port robotic equipment in pediatric surgery.

**Table 1:** Patients’ characteristics and operative data

Patient	1	2	3	4	5
Age (years)	12	4	7	2	9
Gender	Male	Female	Female	Female	Female
Weight (kg)	35	15	27	13	28
Height (cm)	150	103	121	89	135
BMI	15.6	14.2	18.5	16.5	15.4
Cyst type	Ia	Ia	Ia	Ia	Ia
Cyst diameter (cm)	11.8	4.5	4	6.6	4.3
Operative time (min)	230	180	232	220	184
Robotic-assisted operative time (min)	85	60	90	85	70
Blood loss volume (ml)	5	6	10	5	5
Blood vessel, intestine, or pancreas accidental injury	No	No	No	No	No
Intra-abdominal or wound infection	No	No	No	No	No
Cholangitis	No	No	No	No	No
Biliary fistula	No	No	No	No	No
Fasting time (days)	4	5	7	5	3
Postoperative hospitalization time (days)	7	8	14	10	7
Parent satisfaction regarding cosmetic outcomes (SBSES)	4.0	4.0	4.0	5.0	5.0

SBSES, Stony Brook Scar Evaluation Scale.

## Discussion

In this study, we applied robotic-assisted single-port technology to pediatric choledochal cyst resection using the da Vinci Xi system for the first time. While robotic-assisted single-port technology has been utilized in adult robotic-assisted surgery and other pediatric procedures, these operations were typically performed using the da Vinci SP system, which is not specially designed for pediatric patients due to limited abdominal space^[5–8]^.

Recent studies have demonstrated that robot-assisted single-port or single-site surgery can be achieved through various methods. These include pure single-port robots, such as those developed by the Da Vinci Robot Company and the SHURUI single-port robots produced in China[9]. Additionally, traditional multi-port robot systems, like the Da Vinci Si or Xi, can be adapted for single-port procedures by refining the surgical approach^[10–12]^. For instance, Wen Zhang has successfully performed single-port inguinal hernia surgery in children using the Da Vinci Xi system[13]. Currently, the Da Vinci SP system is being utilized in pediatric operating rooms for various procedures, including urological surgeries such as pyeloplasty[14]. And J.M. Smith shows that SP robot-assisted laparoscopic pyeloplasty takes longer but appears comparable with respect to other outcomes[15]. And the cosmetic outcome in a study does not appear to be aesthetically satisfactory, for the incision was not located in the umbilicus[11]. However, these applications are primarily based on small-scale observational studies, with the pediatric patients generally being older[16]. Furthermore, there are no reported observational studies on the use of the Xi robot in the radical surgery of choledochal cysts.

By leveraging the da Vinci Xi system, we enhanced minimally invasive strategies and reduced the number of trocars used in previous robotic-assisted choledochal cyst resections. Currently, there is no dedicated single-port robotic device designed specifically for pediatric use in clinical settings. The da Vinci robotic system, which is the most widely used in clinical practice, can be adapted for single-port technology, offering significant clinical benefits for children. Our previous findings indicate that the robotic-assisted single-port plus one approach does not increase the duration or complexity of the operation. Furthermore, we successfully transitioned to a pure single-port approach in pediatric choledochal cyst resection, demonstrating that it does not prolong operative time compared to multiple-port and adult studies^[17–19]^.

Therefore, our study shows that pure single-port robotic-assisted surgery for the treatment of pediatric choledochal cysts is a safe and effective method. It reduces surgical trauma, does not increase the complexity of the procedure, and does not significantly prolong the surgical time. Given the current absence of single-port robotic devices suitable for pediatric surgery, our technical enhancements with the da Vinci Xi system highlight the potential for integrating single-port technology into pediatric robotic procedures. This not only provides a reliable technical pathway but also offers valuable insights for the future development of single-port robotic equipment in pediatric surgery.

We observed short-term postoperative complications, including accidental injuries to blood vessels, intestines, or the pancreas, as well as intra-abdominal or wound infections, cholangitis, and biliary fistulas. However, a larger patient sample and longer follow-up are necessary to fully evaluate the technique.

To perform single-port laparoscopic surgery and multi-port robotic surgery, surgeons must have sufficient experience and navigate the learning curve. The single-port plus one approach can serve as an intermediate step, facilitating the transition from multi-port to pure single-port techniques and aiding surgeons in mastering the learning curve. The surgeon has completed approximately 20 single-port laparoscopic pediatric choledochal cyst resections and 15 other types of single-port da Vinci robotic surgeries, thereby successfully navigating the learning curve^[20–22]^. Consequently, robotic-assisted single-port pediatric choledochal cyst resection using the da Vinci Xi system is feasible, safe, and effective.

Further studies with larger patient cohorts are needed to assess long-term complications and the learning curve. Additional cases and major prospective multicenter studies are essential to evaluate the feasibility, safety, and efficacy of this technique. As more surgeons adopt the method, it will aid in assessing the learning curve. Future research should also provide detailed data and insights into training requirements and surgeon experience, offering practical guidance for its implementation.

## Conclusion

The robotic-assisted single-port technology using the da Vinci Xi system is safe and effective for pediatric choledochal cyst resection, offering a reliable technical approach and valuable insights for the future application of single-port robotic equipment in pediatric surgery. However, further studies with larger patient cohorts are necessary to assess long-term complications and the learning curve.

## Data Availability

The data analyzed in this study are not publicly available owing to ethical concerns. Further information about the data and technological details for access are available by contacting the corresponding author.

## Research registration unique identifying number (UIN)

This is a retrospective study.

## Supplemental material

Video was submitted as Supplementary Video.

## Declaration

AI was not used in this research and manuscript development.
